# Neurological Manifestations of Coronavirus Disease 2019: A Comprehensive Review and Meta-Analysis of the First 6 Months of Pandemic Reporting

**DOI:** 10.3389/fneur.2021.664599

**Published:** 2021-08-12

**Authors:** Samuel F. Huth, Sung-Min Cho, Chiara Robba, David Highton, Denise Battaglini, Judith Bellapart, Jacky Y. Suen, Gianluigi Li Bassi, Fabio Silvio Taccone, Rakesh C. Arora, Glenn Whitman, John F. Fraser, Jonathon P. Fanning

**Affiliations:** ^1^Critical Care Research Group, The Prince Charles Hospital, Brisbane, QLD, Australia; ^2^Faculty of Medicine, University of Queensland, Brisbane, QLD, Australia; ^3^Neuroscience Critical Care Division, Departments of Neurology, Neurosurgery, and Anaesthesiology and Critical Care Medicine, Johns Hopkins University School of Medicine, Baltimore, MD, United States; ^4^San Martino Policlinico Hospital, IRCCS for Oncology and Neuroscience, University of Genoa, Genoa, Italy; ^5^Princess Alexandra Hospital Southside Clinical Unit, Division of Surgery, Department of Anesthesia, University of Queensland, Brisbane, QLD, Australia; ^6^Department of Medicine, University of Barcelona, Barcelona, Spain; ^7^Intensive Care Services, Royal Brisbane and Women's Hospital, Brisbane, QLD, Australia; ^8^Biomedical Science, Queensland University of Technology, Brisbane, QLD, Australia; ^9^Department of Pulmonary and Critical Care, Institut d'Investigacions Biomediques August Pi I Sunyer, Barcelona, Spain; ^10^Department of Intensive Care, Hôpital Érasme, Brussels, Belgium; ^11^Cardiac Sciences Program, St. Boniface General Hospital Research Center, Winnipeg, MB, Canada; ^12^Section of Cardiac Surgery, Department of Surgery, Max Rady College of Medicine, University of Manitoba, Winnipeg, MB, Canada; ^13^Intensive Care Services, St. Andrew's War Memorial Hospital, UnitingCare, Brisbane, QLD, Australia; ^14^Department of Neurology, Gold Coast University Hospital, Gold Coast, QLD, Australia

**Keywords:** COVID-19, neurological injury, neurological complication, critical care, intensive care

## Abstract

**Background:** There is growing evidence that SARS-Cov-2 infection is associated with severe neurological complications. Understanding the nature and prevalence of these neurologic manifestations is essential for identifying higher-risk patients and projecting demand for ongoing resource utilisation. This review and meta-analysis report the neurologic manifestations identified in hospitalised COVID-19 patients and provide a preliminary estimate of disease prevalence.

**Methods:** MEDLINE, Embase and Scopus were searched for studies reporting the occurrence of neurological complications in hospitalised COVID-19 patients.

**Results:** A total of 2,207 unique entries were identified and screened, among which 14 cohort studies and 53 case reports were included, reporting on a total of 8,577 patients. Central nervous system manifestations included ischemic stroke (*n* = 226), delirium (*n* = 79), intracranial haemorrhage (ICH, *n* = 57), meningoencephalitis (*n* = 13), seizures (*n* = 3), and acute demyelinating encephalitis (*n* = 2). Peripheral nervous system manifestations included Guillain-Barrè Syndrome (*n* = 21) and other peripheral neuropathies (*n* = 3). The pooled period prevalence of ischemic stroke from identified studies was 1.3% [95%CI: 0.9–1.8%, 102/7,715] in all hospitalised COVID-19 patients, and 2.8% [95%CI: 1.0–4.6%, 9/318] among COVID-19 patients admitted to ICU. The pooled prevalence of ICH was estimated at 0.4% [95%CI: 0–0.8%, 6/1,006].

**Conclusions:** The COVID-19 pandemic exerts a substantial neurologic burden which may have residual effects on patients and healthcare systems for years. Low quality evidence impedes the ability to accurately predict the magnitude of this burden. Robust studies with standardised screening and case definitions are required to improve understanding of this disease and optimise treatment of individuals at higher risk for neurologic sequelae.

## Introduction

The neurologic impact of severe acute respiratory syndrome coronavirus 2 (SARS-Cov-2) infection is the subject of widespread study following early reports of significant neurological complications. While neurologic manifestations such as olfactory dysfunction and headache are common with coryza, preliminary reports on SARS-COV-2 infection have frequently identified a host of severe central and peripheral nervous system manifestations in up to 36% of patients, including cerebrovascular accidents, meningoencephalitis, and Guillain-Barré Syndrome (GBS) (1).

Such reports have sparked interest in elucidating the short and longer-term neuropathogenic potential of this virus. Previous coronavirus pandemics, including the severe acute respiratory syndrome (SARS) outbreak in 2002 and the Middle East respiratory syndrome (MERS) outbreak in 2012, demonstrated limited evidence of similarly severe neurologic complications (2, 3). This included scattered reports of stroke, encephalopathy, and neuromuscular dysfunction. Owing to the smaller size of these outbreaks, targeted investigations of these manifestations were scarce, as is evidence regarding their aetiology, incidence, and risk factors. Subsequent animal studies identified significant neuro-invasive potential with both SARS and MERS coronaviruses directly invading brain parenchyma (3, 4). Early human autopsy studies have revealed a similar predilection for SARS-Cov-2, with evidence of cerebrovascular endotheliitis and mixed reports of neuronal invasion in humans (5–7). As the SARS-COV-2 pandemic continues to grow in magnitude, with more than 54 million people infected and 1.3 million deaths worldwide, a thorough investigation of the neurologic manifestations of SARS-COV-2 is vital to identifying risk factors, optimising management, and predicting the long-term impact of the virus (8).

This review presents a timely and comprehensive analysis of available literature pertaining to neurologic manifestations of coronavirus disease 2019 (COVID-19). While substantive efforts have been made to study and comment on select patient cohorts, at the time of writing, this is the first combined meta-analysis and systematic review on the subject. Our primary objective is to offer a comprehensive summary of objective neurologic manifestations identified throughout COVID-19's clinical course. This review focuses on significant neurologic complications, rather than subjective or constitutional symptoms. As such, symptoms including headache, malaise, gustatory/olfactory dysfunction, and headache were excluded, having been reviewed elsewhere (9).

## Methods

This systematic review and meta-analysis was conducted using the Joanna-Briggs Institute (JBI) Reviewer's Manual for Systematic Reviews of Literature, and in accordance with Preferred Items for Systematic Review and Meta-analysis (PRISMA) guidelines (10, 11). A completed PRISMA checklist can be found in Supplementary Materials.

### Search Strategy

MEDLINE, Embase and Scopus were searched for items published from inception to the 17th July 2020. No restrictions were placed on article type. The literature search strategy included a combination of topic headings and key words structured to include studies which focused solely on neurologic manifestations and broader observational studies which reported a variety of symptoms and complications, including neurological. The basic structure of the search terms was (*Covid-19 OR sars-cov-2 OR 2019-ncov) AND (injury OR complication OR manifestation OR presentation) AND (neurologic injury/exp OR brain injury/exp OR neurologic/exp)*. A full list of expanded search terms can be found in Supplementary Materials. To ensure governmental reports and articles in pre-print were not unduly excluded, targeted searching was also conducted using Google Scholar and medRxiv, respectively.

All entries identified through literature searching were exported to Endnote X9 for screening. Duplicate references were removed automatically. Screening of all articles was performed concurrently by two reviewers (SFH and JPF), with discrepancies resolved through discussion with the other authors.

### Selection Criteria

Research papers/reports were included when they met two criteria; (1) the study population included patients diagnosed with COVID-19 by laboratory real-time polymerase chain reaction (RT-PCR) confirmation; and, (2) the study described the occurrence of neurologic manifestations in patients as either a primary or secondary endpoint. A broad and inclusive definition was employed for neurologic manifestations, including complications identified on presentation as well as during the clinical course of COVID-19. All quantitative and qualitative research was included, including case reports, case series, case-control studies, cohort studies, cross-sectional studies, and randomised controlled trials. Letters and other forms of correspondence were included if they reported original data.

Articles were principally excluded if they met either of two criteria; (1) the study solely reported subjective or constitutional symptoms (namely fatigue, dizziness, drowsiness, headache, and reduced smell/taste) that failed to meet formal criteria for diagnosing a neurologic condition, or (2) the study was one of multiple studies that reported results from the same cohort of patients, or a subset of a larger population reported elsewhere. When exclusion criterion 2 was met, only the largest or most recent study which reported relevant neurologic endpoints was included.

In addition, neurologic manifestations resulting from procedural complications unrelated to COVID-19 (invasive central/arterial line placement, drug toxicity, proning) were excluded. Studies were also excluded where an English language article or translation was unavailable.

### Data Extraction

Data were extracted in duplicate by two reviewers (SFH and JPF) using a standardised data extraction tool. Any discrepancies were resolved by a third reviewer (DH). The data extraction tool included descriptive variables (publication date, study region, study design, sample size, COVID-19 diagnostic criteria/method, patient age, patient sex), and variables pertaining to neurologic complications (type of manifestation, diagnostic criteria/definition, time of diagnosis/screening, manifestation severity score/scale). If possible, pertinent variables associated with complications were extracted for inclusion in meta-analysis.

### Data Analysis/Synthesis

Descriptive data pertaining to the types of complication, diagnostic methods, clinical features, and patient demographics were reported in narrative and tabular form. Numerical data on the prevalence of neurologic manifestations were collated for quantitative analysis.

Meta-analysis was conducted for all identified studies which reported the point or period prevalence of neurologic manifestations. This could only be calculated for manifestations reported across multiple studies employing consistent diagnostic and inclusion criteria. OpenMeta Analyst was used to calculate pooled prevalence and generate Forest plots (12). A random effects model was used, due to significant heterogeneity in the sampling methodology and populations between studies.

### Data Analysis/Synthesis

The protocol for assessing study quality/risk of bias varied, depending on study architecture. For case-control or cohort studies, a COVID-19 adapted Newcastle-Ottawa Scale (NOS) was used (13, 14). A modified, 8-item NOS proposed by Murad and colleagues was used to assess case reports and case series (15).

## Results

**Figure 1:**
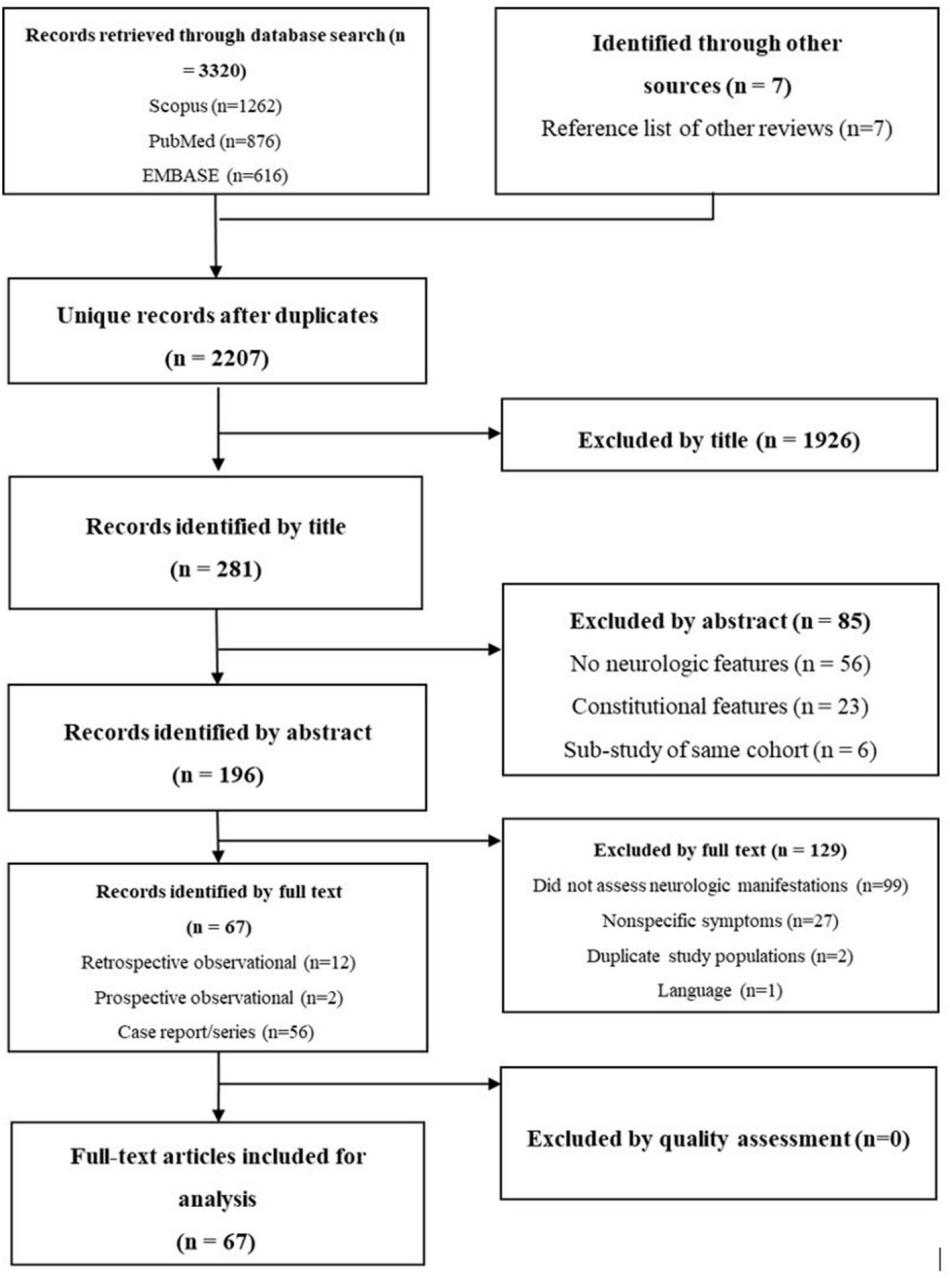
PRISMA flowchart for screening of literature.

The literature search yielded 2,207 unique entries. The main reasons for excluding entries by title and abstract were the lack of reporting of neurologic manifestations and the sole reporting of subjìtive coryzal and constitutional symptoms. Figure 1 provides a PRISMA flowchart depicting article screening.

A total of 67 studies were included for full-text review. These studies reported neurologic manifestations over a total population of 8,577 patients with laboratory-confirmed COVID-19 exposure. The diagnostic method for COVID-19 was consistent across all 67 studies, consisting of nasopharyngeal swab with quantitative real-time polymerase chain reaction (RT-PCR). The geographic distribution of centres was diverse, with studies based in Turkey, Saudi Arabia, USA, China, United Arab Emirates, France, Singapore, Italy, Spain, the Netherlands, and the UK.

Fourteen observational cohort studies and 53 case studies were included. Pertinent features regarding the cohort studies are outlined in Table 1. Sample size varied considerably, with the smallest study including 50 patients and the largest 3,218. Thirteen of the cohort studies included patients of all ages, with one study solely investigating paediatric patients (16). Patient age ranged from 12 weeks to 105 years, with a majority male population (58% overall). Three studies solely investigated ischemic stroke, and one study focused solely on thromboembolic events (including stroke). Two studies solely reported neuroimaging findings in the context of COVID-19. The remaining studies evaluated any diagnosed neurologic manifestations. Only one of the studies included a screening tool with standardised definitions for neurologic manifestations, while the remaining studies relied on local medical diagnosis/reporting (29).

**Table 1:** Summary of cohort studies investigating neurologic manifestations in COVID-19.

**Author**	**Date**	**Country**	**Type**	**Population**	**COVID diagnosis criteria**	**Sample size**	**Sex - male (%)**	**Mortality rate**	**Average Age**	**Manifestations reported**
Abdel-mannan (16)	1/07/2020	UK	Retrospective Cohort Study	Hospitalised COVID-19 Patients	Nasopharyngeal RT-PCR or IV IgG	50	48 (96%)	N.S.	12 [RANGE: 8–15]	Non-specific encephalopathy
Helm (17)^*^	4/06/2020	France	Retrospective Cohort Study	COVID-19 ICU Patients	Nasopharyngeal RT-PCR or IV IgG	58	N.S.	N.S.	63	Dysexecutive syndrome, Delirium, Corticospinal tract syndrome, Leptomeningeal enhancement, Ischemic stroke
Helm (18)^*^	4/06/2020	France	Prospective Cohort Study	COVID-19 ICU Patients	Nasopharyngeal RT-PCR or IV IgG	150	122 (81%)	8.7%^*^	63 [IQR: 53–71]	Ischemic stroke
Jain (19)	19/05/2020	USA	Retrospective Cohort Study	Hospitalised patients	Nasopharyngeal RT-PCR or IV IgG	3,218	60.7%	N.S	64 [RANGE: 2w−105y]	Ischemic stroke, Intracerebral haemorrhage, Encephalitis
Klok (20)	30/04/2020	Netherlands	Retrospective Cohort Study	COVID-19 ICU Patients	Nasopharyngeal RT-PCR or IV IgG	184	139 (76%)	22%^*^	63 [STD: 12]	Ischemic stroke
Kremer (21)	16/06/2020	France	Retrospective Cohort Study	Hospitalised COVID-19 Patients with Neurologic Symptoms	Nasopharyngeal RT-PCR or IV IgG	190	N.S.	N.S.	N.S.	Intracerebral haemorrhage
Li/Mao (1, 22)	1/04/2020	China	Retrospective Cohort Study	Hospitalised COVID-19 Patients	Nasopharyngeal RT-PCR or IV IgG	219	89	N.S	53.5 [STD: 15.9]	Ischemic stroke, intracerebral haemorrhage, delirium, seizure
Lodigiani (23)	16/04/2020	Italy	Retrospective Cohort Study	Hospitalised COVID-19 Patients	Nasopharyngeal RT-PCR or IV IgG	388	264 (68%)	26%	66 [IQR: 55–85]	Ischemic stroke
Lu (24)^**^	6/04/2020	China	Retrospective Cohort Study	Hospitalised COVID-19 Patients	Nasopharyngeal RT-PCR or IV IgG	304	59.9%	3.30%	44 [RANGE: 33–59]	Seizure, cerebrovascular injury
Merkler (25)	21/05/2020	USA	Retrospective Cohort Study	Hospitalised COVID-19 Patients	Nasopharyngeal RT-PCR or IV IgG	2,132	1,173 (55%)	-	62 [IQR: 48–75]	Ischemic stroke
Romero-Sanchez (26)	1/06/2020	Spain	Retrospective Cohort Study	Hospitalised COVID-19 Patients	Nasopharyngeal RT-PCR or IV IgG	841	471 (56%)	23.40%	66.4	Ischemic stroke, intracerebral haemorrhage, delirium, encephalitis, ADEM, neuropathy
Scullen (27)	19/05/2020	USA	Retrospective Cohort Study	COVID-19 ICU Patients	Nasopharyngeal RT-PCR or IV IgG	76	52%	4%	N.S.	Ischemic stroke, intracerebral haemorrhage, non-specific encephalopathy
Xiong (28)^**^	17/07/2020	China	Retrospective Cohort Study	Hospitalised COVID-19 Patients	Nasopharyngeal RT-PCR or IV IgG	917	55%	3.9	48.7 [STD: 17.1]	Ischemic stroke, delirium
Vartharaj (29)	25/06/2020	UK	Prospective Cohort Study	Hospitalised COVID-19 Patients with Neurologic Symptoms	Nasopharyngeal RT-PCR or IV IgG	125	48%	N.S.	71 [IQR: 58–79]	Ischemic stroke, intracerebral haemorrhage, encephalitis

* *Both Helm studies are based on the same population*.

** *The study by Xiong assesses the same patients reported by Lu and colleagues*.

*N.S., not specified; ICU, intensive care unit; IV, intravenous; IgG, immunoglobulin G; ADEM, acute disseminated encephalomyelitis*.

Of the 67 articles, 31 were rated as low quality, 35 moderate quality, and one high quality. Overall, articles lacked clarity regarding the methods used to screen for neurologic cases. Articles which included robust descriptions of the methodology for case identification and diagnostic criteria were categorised as moderate or high quality. A full summary of article quality can be found in Supplementary Materials.

### Central Nervous System Manifestations

Central nervous system (CNS) manifestations included ischemic stroke in a total of 226 patients, delirium in 79 patients, intracranial haemorrhage in 57 patients, meningoencephalitis in 13 patients, acute demyelinating encephalomyelitis (ADEM) in two patients, and seizure in one patient.

### Ischemic Stroke

**Figure 2:**
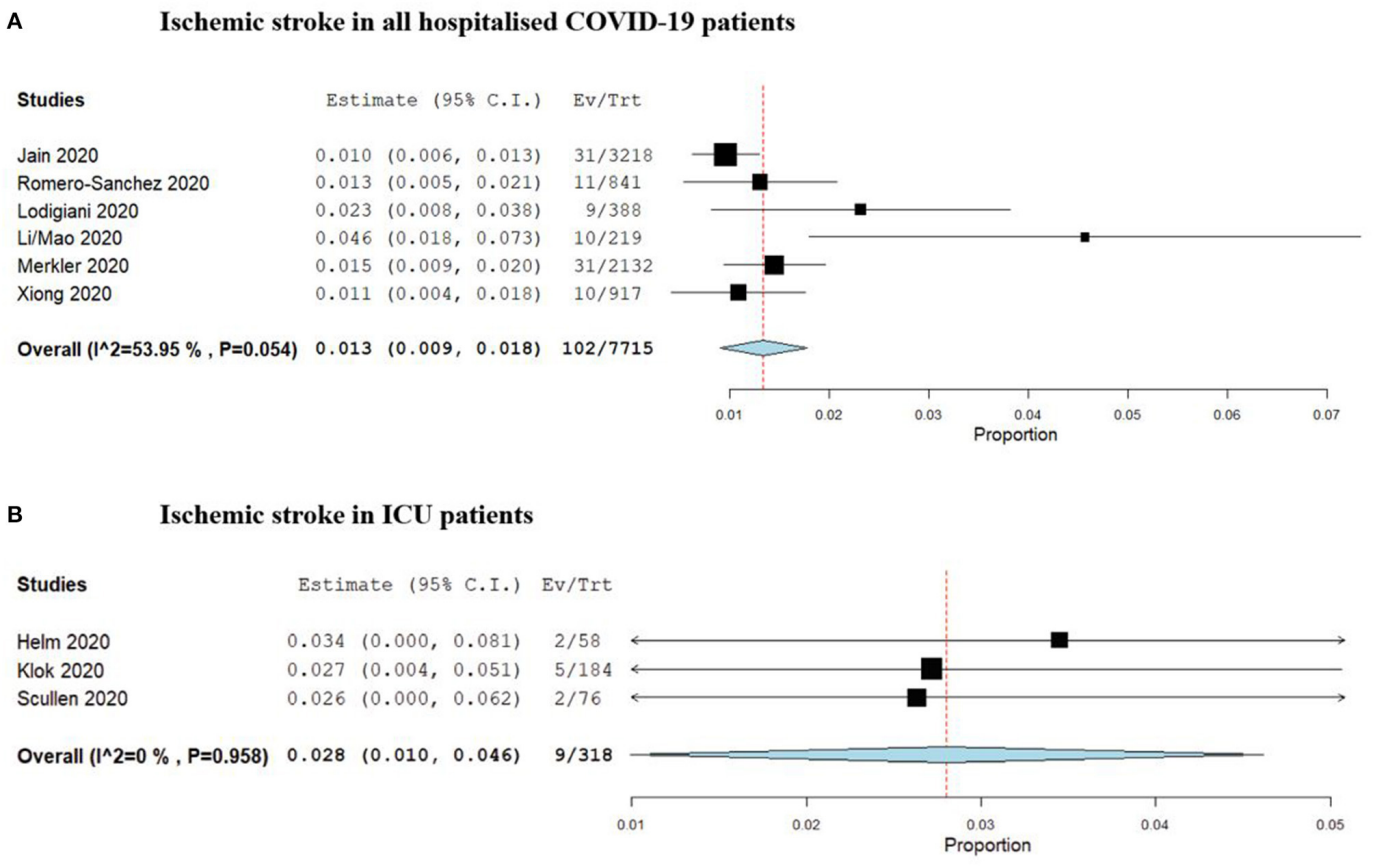
Forrest plots of period prevalence of **(A)** Ischaemic stroke in all hospitalised COVID-19 patients, and **(B)** Ischaemic stroke in patients requiring ICU admission.

Mao and colleagues were the first to report period prevalence of ischemic stroke during the clinical course of hospitalised COVID-19 patients in a multicentre trial involving 158 patients in Wuhan, China. A separate report from Helms and colleagues reporting the rate in a select group of 58 ICU-admitted patients in France (1, 17, 18). Two similar studies followed (23, 28). One study by Jain and colleagues was the largest, reporting the rate of ischemic stroke, based on imaging findings, in 3,218 patients within a hospital system in New York (19). The pooled period prevalence of ischemic stroke from identified studies is 1.3% [95% CI: 0.9–1.8%, 102/7,715] for all hospitalised COVID-19 patients over their clinical course, and 2.8% [95% CI: 1.0–4.6%, 9/318] for COVID-19 patients admitted to an intensive care unit (ICU). This is summarised in Figure 2. Severity of illness was associated with a higher rate of stroke, as was the presence of comorbid condition and increased age (18, 22). Specific details pertaining to infarct number, volume and distribution were not reported, nor were the methods of management.

The earliest reports of ischemic stroke in COVID-19 came from multiple case reports in older adult patients with concomitant cardiovascular comorbidities, which included atrial fibrillation and coronary artery disease. Most ischemic strokes were reported in patients over 60 years of age, except in a case series of younger stroke patients at a single New York centre which identified stroke in six patients under the age of 55 (30). Multiple studies independently identified associations between ischemic stroke and more severe respiratory infection, with higher incidence rates in critically ill and older patients.

The average time to ischemic stroke occurrence after admission to hospital was 10 days (range 0–33). The average time to diagnosis was higher in ICU patients. In four patients, ischemic stroke was the presenting complaint on admission, though this was unreported in most studies (31). Importantly, all cohort studies solely reported stroke identified during the patient's clinical course without follow-up beyond discharge. An unreported number of patients in these studies were still in ICU at the time of data analysis (1, 22).

In addition to ischemic stroke, some reports of cerebral venous sinus thrombosis (CVST) have emerged. A male patient aged in their 80s acquired CVST as well as bilateral large territory brain infarcts several days after COVID-19 diagnosis (32). Blood analysis showed elevated inflammatory markers. Another patient aged in their 50s presented to hospital with impaired consciousness and was found to have CVST on CT. The patient was subsequently diagnosed with COVID-19 (33).

### Intracranial Haemorrhage

**Figure 3:**
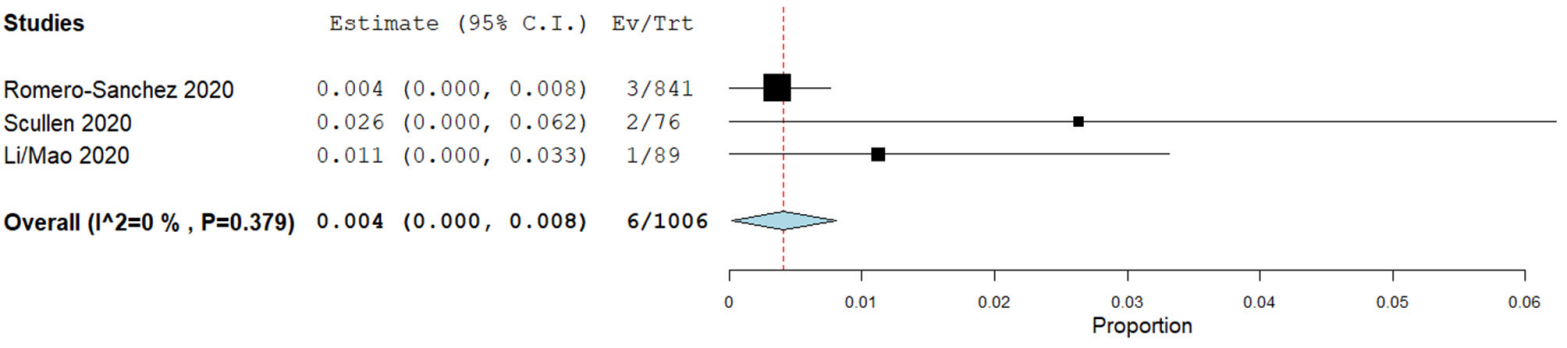
Forrest plot of period prevalence of intracranial haemorrhage in hospitalised COVID-19 patients.

Three cohort studies estimating the period prevalence of ICH during the hospital stay of COVID-19 patients were identified (1, 22, 26, 27). The pooled prevalence of ICH (Figure 3) was estimated at 0.4% [95% CI: 0–0.8%, 6/1,006], with significant variability in rates and population sizes between studies (0.4–2.6%, 76–841 patients). The largest study identified three such patients in a cohort of 841 (0.4%), which was the lowest rate reported (26). Specific details regarding the risk factors, location and nature of the haemorrhages were not provided.

Multiple case reports of ICH were identified in the literature (Supplementary Materials). Patient age ranged from 38 to 67, with most patients in their fifties. The time to onset of neurologic symptoms was highly variable, with ICH most often diagnosed after two weeks of illness, but the earliest presentation after three days of the onset of coryzal symptoms (34). In six of the eight cases, ICH occurred following ICU admission and invasive ventilation. In one case, the patient presented to hospital with neurologic deficit (34).

Multiple variants of intracerebral haemorrhage were noted, ranging from large focal haemorrhage with a mass effect to multiple microhemorrhages associated with vasogenic oedema. All patients underwent computed tomography (CT) imaging with Magnetic Resonance Imaging (MRI) used in cases where CT revealed lesions with unclear aetiology, as in three cases of haemorrhagic posterior reversible encephalopathy syndrome (PRES) and one case of bilateral thalamic microhemorrhages (34–36). One of the ICH patients was undergoing extracorporeal membrane oxygenation (ECMO) therapy at the time of this complication, identified by unilateral facial weakness and dysarthria in the ICU (37).

### Encephalitis & Meningitis

Few cohort studies have measured the prevalence of encephalitis. The only case reported in a large observational study of all COVID-19 hospitalised patients was by Romero-Sanchez and colleagues, who reported a single patient in 841 cases (0.1%) (26). A prospective study by Vartharaj and colleagues that investigated all COVID-19 patients with neurologic manifestations identified seven cases in 153 patients (4.5%) (29).

Four case studies have reported COVID-19 patients developing encephalitis (Supplementary Materials). All these cases involved males, aged 28–40. Three of these four patients presented with mild cognitive impairment or altered mental status (38–40). The diagnosis of SARS-COV-2 encephalitis was principally made after other conditions were excluded and the patient responded to antiviral therapy. Only one patient was reported to be positive for SARS-COV-2 RNA RT-PCR in cerebrospinal fluid (CSF), with unremarkable CSF analysis in other patients. One patient developed neurologic features three 3 days post admission (41). The diagnosis of SARS-COV-2 encephalitis was principally made after other conditions were excluded and the patient responded to antiviral therapy. Only one patient was reported to be positive for SARS-COV-2 RNA RT-PCR in cerebrospinal fluid (CSF), with CSF analysis in other patients yielding no notable findings (39).

Diffusion weighted imaging (DWI) MRI identified hyper intensities in the ventricular walls of one patient with concurrent FLAIR hyper intensities within the brain parenchyma (39). Rhombo-encephalitis was diagnosed in one patient via identification of T2 hyper intensities in the brain stem and cervical spine (41).

Symptoms of encephalitis were highly variable, ranging from gait abnormalities and visual disturbance to depressed consciousness. Signs of meningism were present in two patients, including nuchal rigidity, Kernig's sign, and Brudzinski's sign (39, 40). One patient recovered fully with no residual neurologic deficits, one patient was discharged with ongoing gabapentin therapy, one patient was still in the ICU at the time of reporting, and outcomes for one patient were not reported.

### Delirium

Three studies reported the period prevalence of delirium in hospitalised COVID-19 patients. Significant heterogeneity in diagnostic criteria, sample size, and screening methodology precluded pooled meta-analysis. Helms and colleagues reported the highest prevalence, identifying delirium in 26/40 (65%) critically ill patients using the confusion assessment method for the intensive care unit (CAM-ICU) screening tool (17). It is unclear, however, whether screening was routine or selectively targeted at patients with signs of neurocognitive dysfunction. A second study by Romero-Sanchez and colleagues identified delirium in 79/841 (9.3%) hospitalised COVID-19 patients (26). Delirium was more common in patients requiring an ICU admission. Xiong and colleagues reported seven cases of delirium in 917 hospitalised patients (0.7%) (28). Across all three studies, the threshold for screening was unclear and Xiong and Romero-Sanchez failed to report on whether the screening tool they used was a standardised or non-standardised (26, 28).

Three case reports of delirium were identified (42–44). All three involved male patients aged 70 or older. Two of these patients presented to hospital with acute confusion and unusual behaviour, while the other presented after being found on the ground following a fall. On admission, all three patients reported no significant respiratory symptoms. COVID-19 was confirmed in one patient through routine nasopharyngeal swab, while testing in the other two was prompted by chest CT findings consistent with COVID-19 respiratory disease.

Individualised investigations for delirium were not reported in observational series, however broad trends in imaging findings and neurologic investigations offer some insight. In the study by Helms *et al*., brain MRI identified leptomeningeal enhancement in eight patients and bilateral frontotemporal hypoperfusion in 11 patients. It is unknown whether this was the result of direct neuroinvasion, as lumbar puncture was scarcely performed with no comparison with imaging findings. EEG identified non-specific changes in one patient (17). Xiong *et al*. did not report any brain CT findings relating to delirium in 28 patients imaged, and brain MRI was not performed. Lumbar puncture results were negative in one patient tested (28). Romero-Sanchez and colleagues reported negative brain MRI and EEG findings in two patients with delirium and pyramidal signs. No lumbar puncture was performed in their cohort (26).

A definitive cause of delirium was not identified in any case reports (42–44). All patients were found to have normal oxygen saturation at the time of onset of delirium and two patients underwent brain CT which identified no intracranial pathology. Lumbar puncture was not performed or was refused.

Across all reports, SARS-Cov-2 infection was suggested as the underlying cause of delirium in the absence of definitive diagnostic evidence of other pathologies. Two mechanisms were suggested; direct neuroinvasion and secondary systemic effects of COVID-19. There was insufficient data in severely ill patients to discern between direct effects of the virus and encephalopathy related to critical illness, except in the report by Xiong and colleagues which analysed patients with known complications separately.

### Seizures

Seizures were identified in two patients by Li/Mao in a population of 219 hospitalised COVID-19 patients (0.9%) (1, 22). Both patients were critically ill ICU patients. Lu and colleagues sought to specifically investigate the occurrence of seizures in multiple centres in the Sichuan region of China, but identified only one patient with seizure-like symptoms, and no cases meeting clinical or EEG criteria for seizures amongst a total population of 304 patients (24). Neither of these studies explicitly screened for seizures using continuous EEG monitoring, instead retrospectively reporting on the occurrence of a seizure as noted by the treating team. Two case reports have been published in the literature describing COVID-19 patients for whom the presenting complaint was a motor seizure (Supplementary Materials). One of these patients had a history of post-encephalitic epilepsy, while the other had no history of epilepsy or seizures (45).

### Acute Disseminated Encephalomyelitis

Two cases of ADEM have been reported in the literature (Supplementary Materials) (46, 47).

The first patient was an elderly male who was admitted to hospital to undergo CABG for underlying coronary artery disease (46). Six days post operatively, the patient developed worsening respiratory failure and kidney injury necessitating intubation and admission to the ICU. He died 5 days later. Post-mortem examination revealed multiple demyelinating lesions in the sub-cortical white matter associated with microhaemorrhages.

The second patient was an elderly female who was admitted following a two-week flu-like illness which was confirmed by serum IgG testing to be COVID-19 (47). She had a background of monoclonal gammopathy of unknown significance. She was admitted 2 weeks after the resolution of her flu-like symptoms with bilateral visual impairment. MRI revealed multiple T1 gadolinium-enhancing lesions in the spinal cord and optic nerves. Lumbar puncture identified lymphocytic pleocytosis and positive RT-PCR results for SARS-COV-2 viral RNA. She was managed with high-dose IV methylprednisolone and IV immunoglobulins. She recovered visual acuity 2 weeks later.

### Peripheral Nervous System Manifestations

Peripheral nervous system manifestations included GBS and its variants (Miller Fisher Syndrome and others) in 21 patients, and other neuropathic syndromes including facial nerve palsy and peripheral motor neuropathy in three patients.

### Guillain-Barre Syndrome & Miller Fisher Syndrome

The period prevalence of GBS in COVID-19 patients has not been studied in the literature. Toscano and colleagues are the only authors to offer a preliminary estimate, identifying five COVID-19 patients with GBS during a period during which 1,000–1,200 COVID-19 patients were treated (0.4–0.5% of cases) (48). However, GBS and Miller Fisher Syndrome (MFS) have been described in case reports and small case series (Supplementary Materials).

A total of 15 case reports/series encompassing 21 patients, have been published. The age of patients identified in these reports varied from 23 to 77 (median age 55), demonstrating male predominance (15/21, 71%). The initial clinical presentation of COVID-19 patients with GBS was highly variable, several patients presenting with isolated neurologic symptoms several weeks post infection, and many others developing neurologic symptoms during inpatient treatment of severe infection. The onset of neurologic symptoms typically occurred 2–3 weeks following the onset of respiratory symptoms.

Investigations for GBS typically included brain and spine imaging, as well as CSF analysis, neurophysiology studies, and serum antibody testing. Neuroimaging was principally used to exclude other neurologic causes; but it also identified cranial nerve inflammation in one patient and inflammation of the dorsal root ganglia in another (48, 49). CSF and serum analysis chiefly identified albumin cytogenic dissociation and oligoclonal bands, respectively. The individual investigations and respective results for each patient are summarised in Supplementary Materials.

All patients with GBS were treated with intravenous IgG (IVIG), with three patients also undergoing plasmapheresis (48, 50). Two papers did not explicitly report patient outcomes, five patients were reported to recover fully during inpatient treatment, 10 were reported to recover partially with residual deficits or ongoing therapy, and four were reported to be unresponsive to therapy.

### Other Peripheral Neuropathy

Several case reports of non-specific peripheral neuropathy have been published in the literature (Supplementary Materials).

Abdelnour and colleagues described an elderly male who presented with bilateral lower limb weakness, areflexia, and an ataxic gait, but no acute respiratory symptoms (51). Four days post admission, this patient developed respiratory symptoms and was diagnosed with COVID-19. No sensory deficits or other neurologic symptoms were identified. His lower limb motor deficit resolved spontaneously over 3 weeks. Dinkin reported on two patients (52). The first was a middle-aged male with cranial nerve neuropathy. This patient responded to treatment with IVIG in 3 days. The other patient was an elderly female patient who presented with painless diplopia and eye abduction failure. No specific treatment was administered to this patient. Goh and colleagues reported a case of Bell's palsy in a young adult male thought to be caused by SARS-COV-2 (53).

## Discussion

This review identified 67 studies that specifically evaluated the neurological complications of hospitalised COVID-19 patients. Previous reviews have described the range of neurologic manifestations reported in the literature and attempted to clarify case definitions for future studies. Similarly, prior reviews have focused on presenting neurologic symptoms like ageusia and anosmia. To our knowledge, this is one of the first papers to describe the results of a comprehensive systematic review and meta-analysis to estimate the period prevalence of neurologic manifestations in hospitalised COVID-19 patients. The major conclusion of our review is that current evidence regarding most manifestations is limited to retrospective descriptive studies. Pooled prevalence could only be estimated for ischemic stroke and intracranial haemorrhage, due to the paucity of quality evidence for other complications. Accurate description of the prevalence, risk factors, and management strategies for neurologic manifestations in COVID-19 is essential for determining the foreseeable risk and burden to patients and the broader healthcare system, and for improving diagnosis and management.

In previous coronavirus pandemics, namely SARS and MERS, few studies were published which estimated the prevalence of neurologic manifestations. In one SARS study based in Singapore, five of 206 hospitalised patients (2.4%) acquired large territory ischemic stroke (54). Most of these patients already had pre-existing coagulopathy and cardiovascular diseases which possibly skewed results. This prevalence rate is similar to estimates reported for SARS-Cov-2, with our analysis identifying a pooled prevalence of ischemic stroke of 1.3% [95% CI: 0.9–1.8%, 102/7,715] in all hospitalised patients and 2.8% [95% CI: 1.0–4.6%, 9/318] among patients requiring ICU admission.

The rate of seizure identified so far in hospitalised SARS-Cov-2 patients is relatively low, with few case series reported. Early estimates identified in this review were 0.9% in one study of 841 patients and zero cases in a study of 304 patients (24, 26). Of note, neither of these studies explicitly screened for seizures, only reporting it where incidentally observed by the treating team. In a previous MERS study, six of 70 patients admitted to hospital experienced at least one seizure (55). All six patients were critically ill, with an ICU rate in this study population of 70%. Application of robust screening methodology may yield a higher rate of detection in COVID-19 patients.

Delirium is poorly reported in the literature. While “confusion” or “encephalopathy” were reported for multiple studies, neurocognitive assessment or the use of a systematic delirium screening tool to confirm delirium was largely absent. Using CAM-ICU, Helms and colleagues identified delirium in 26 of 40 (65%) critically ill patients (17). Two larger studies with unreported screening methodology identified rates of confusion of 0.7 and 9.3% (26, 28). In MERS, “confusion” was reported in 16 of 70 patients (22%). However, without consistent screening methodology and case definitions there is no way to compare these rates. Across multiple COVID-19 studies, CAM-ICU defined-delirium has been reported to have an incidence of 45–87%, depending on the severity of illness and level of sedation or supportive therapy (56). In this context, a proportion of 65% in mechanically ventilated patients with disseminated viral infection seems reasonable.

The identification of encephalitis was reported to be a rare complication. While multiple reports of non-specific encephalopathy and confusion have been published, the limited diagnostic options for viral encephalitis may contribute to its low rate in currently published studies. SARS-Cov-2 RNA has only been detected in the CSF of one patient in the literature, with a previous study revealing no positive samples in 578 COVID-19 CSF samples (57). While evidence of direct neuronal invasion has been identified, autopsy studies have offered preliminary evidence that neuroinvasion may largely be haematogenous or retrograde with minimal viral load in CSF (5–7).

Acute demyelinating encephalomyelitis (ADEM), Guillain-Barre syndrome (GBS), and Miller Fisher variant (MFV) were identified in multiple patients. ADEM is known to be a very rare complication of disseminated viral infection, so its occurrence in the current SARS-COV-2 pandemic is to be expected. The multiple reports of GBS and MFS appear to confirm that immune-mediated polyneuropathy is a possible complication of COVID-19, with the disease pattern and treatment typical of post-infective GBS. The rate of GBS in COVID-19 patients reported by Toscano and colleagues is exceedingly high (0.4–0.5%), relative to a previous report which identified just nine cases in 30,000 patients (0.03%) infected with Campylobacter jejuni over a 9-year period (48, 58). This disparity likely reflects how Toscano and colleagues reported on the proportion of hospitalised patients with this manifestation, rather than its proportion among all laboratory confirmed COVID-19 patients over the period of observation.

The overall findings of this review therefore indicate that severe neurologic manifestations are associated with COVID-19 at rate which is similar to other infective illnesses and previous coronavirus pandemics. While early reports indicated exceedingly high rates of complications amongst critically ill COVID-19 patients, it is likely that the true rates of stroke, haemorrhage, and delirium are comparable to other critical illnesses and previous coronavirus pandemics. Currently, there is insufficient published evidence to estimate the prevalence of many central and peripheral nervous system manifestations. This is due both to limited data availability and the rarity of many of these complications, such as GBS and encephalitis.

While the rate of complication is not as high as initially suspected the neurological burden is still projected to be substantial considering the magnitude of the pandemic. The authors of a previous review estimated that thousands of COVID-19 patients should be expected to experience severe neurologic manifestations (59). They predicted that the total number of cases with neurological manifestations would ultimately be between 4,213 and 17,408 based upon the rates of complications reported for SARS and MERS. Of note, this prediction did not include stroke as a CNS complication, which, when included, would likely increase this number dramatically.

The major limitation of the current review relates to the quality of evidence identified. As stated previously, most of the articles that we analysed are of moderate-to-poor quality, largely due to limited reporting of screening methodology, case definitions, and cohort geography/distribution. Two papers had to be excluded for reporting identical outcomes in populations, which had been reported elsewhere in the literature. Inferring broader estimates of prevalence from multiple studies of questionable quality is, itself, of questionable validity. Nonetheless, observed agreement between multiple individual studies may indicate that these estimates are reasonable. An additional limitation is the period of patient inclusion. Emerging evidence regarding post-COVID neurologic symptoms necessitates further review of studies reporting manifestations beyond the hospitalisation period.

## Conclusions

Current evidence surrounding the prevalence, risk factors, and management of severe neurologic manifestations in hospitalised COVID-19 patients is scarce and generally of low to moderate quality. Estimating the true burden of the SARS-Cov-2 pandemic will require a systematic approach to data analysis with clear case definitions and controlled screening methodology. Thus, far early evidence and preliminary meta-analysis indicate that the neurologic burden of the SARS-Cov-2 pandemic is likely to be high, particularly in the form of cerebrovascular accidents and severe neuropathic syndromes conveying residual neurologic impairment and the need for ongoing management. This finding, combined with reports of neurologic symptoms as isolated presenting features of COVID-19, demonstrate an inescapable need to acknowledge and better understand the link between SARS-Cov-2 infection and patient neurology.

## Data Availability Statement

The original contributions presented in the study are included in the article/Supplementary Material, further inquiries can be directed to the corresponding author.

## Author Contributions

JPF and SH conceived and designed study. All authors contributed to the refinement of methodology, drafting and final approval of the manuscript. Article screening and data extraction were principally completed by SH and JPF with assistance from DH.

## Conflict of Interest

The authors are all members of the COVID-19 Critical Care Consortium Neurology sub-committee. RA has received an unrestricted educational grant from Pfizer Canada Inc. and honoraria from Mallinckrodt Pharmaceutical, Abbott Nutrition and Edwards Lifesciences that are. GW received funding from the Data Safety Monitoring board of Cytosorbent and Cellphire. GLB receives grant support from University of Queensland, Wesley Medical Research, The Prince Charles Hospital Foundation, The Health Research Board of Ireland, Biomedicine International Training Research Program for Excellent Clinician-Scientists, European Union's Research and Innovation Program (Horizon 2020), and La Caixa Foundation. GLB and JFF have received research support from Fisher & Paykel. The remaining authors declare that the research was conducted in the absence of any commercial or financial relationships that could be construed as a potential conflict of interest.

## Publisher's Note

All claims expressed in this article are solely those of the authors and do not necessarily represent those of their affiliated organizations, or those of the publisher, the editors and the reviewers. Any product that may be evaluated in this article, or claim that may be made by its manufacturer, is not guaranteed or endorsed by the publisher.
