# Supplementary material for: Neurological Manifestations of Coronavirus Disease 2019: A Comprehensive Review and Meta-Analysis of the First 6 Months of Pandemic Reporting
Source: Front Neurol. 2021 Aug 12;12:664599. doi: 10.3389/fneur.2021.664599 (PMC8387564; doi:10.3389/fneur.2021.664599)
Supplement: Supplementary file 1 [file Table_1.DOCX]

Supplementary Material

**Supplementary Materials**

**Item 1:** PRISMA Checklist

**Item 2:** Search terms

**Item 3:** Case reports

**Item 4:** Quality assessment

**Supplementary Item 1: PRISMA Checklist**

| **Section/topic** | **#** | **Checklist item** | **Reported on page** |
| --- | --- | --- | --- |
| **TITLE** | | |  |
| Title | 1 | Identify the report as a systematic review, meta-analysis, or both. | 1 |
| **ABSTRACT** | | |  |
| Structured summary | 2 | Provide a structured summary including, as applicable: background; objectives; data sources; study eligibility criteria, participants, and interventions; study appraisal and synthesis methods; results; limitations; conclusions and implications of key findings; systematic review registration number. | 4 |
| **INTRODUCTION** | | |  |
| Rationale | 3 | Describe the rationale for the review in the context of what is already known. | 5 |
| Objectives | 4 | Provide an explicit statement of questions being addressed with reference to participants, interventions, comparisons, outcomes, and study design (PICOS). | 5 |
| **METHODS** | | |  |
| Protocol and registration | 5 | Indicate if a review protocol exists, if and where it can be accessed (e.g., Web address), and, if available, provide registration information including registration number. | 6 |
| Eligibility criteria | 6 | Specify study characteristics (e.g., PICOS, length of follow-up) and report characteristics (e.g., years considered, language, publication status) used as criteria for eligibility, giving rationale. | 6 |
| Information sources | 7 | Describe all information sources (e.g., databases with dates of coverage, contact with study authors to identify additional studies) in the search and date last searched. | 6 |
| Search | 8 | Present full electronic search strategy for at least one database, including any limits used, such that it could be repeated. | Sup. mat |
| Study selection | 9 | State the process for selecting studies (i.e., screening, eligibility, included in systematic review, and, if applicable, included in the meta-analysis). | 6 |
| Data collection process | 10 | Describe method of data extraction from reports (e.g., piloted forms, independently, in duplicate) and any processes for obtaining and confirming data from investigators. | 7 |
| Data items | 11 | List and define all variables for which data were sought (e.g., PICOS, funding sources) and any assumptions and simplifications made. | 7 |
| Risk of bias in individual studies | 12 | Describe methods used for assessing risk of bias of individual studies (including specification of whether this was done at the study or outcome level), and how this information is to be used in any data synthesis. | 7 |
| Summary measures | 13 | State the principal summary measures (e.g., risk ratio, difference in means). | 6/7 |
| Synthesis of results | 14 | Describe the methods of handling data and combining results of studies, if done, including measures of consistency (e.g., I^2^) for each meta-analysis. | 6/7 |
| Risk of bias across studies | 15 | Specify any assessment of risk of bias that may affect the cumulative evidence (e.g., publication bias, selective reporting within studies). | 7 |
| Additional analyses | 16 | Describe methods of additional analyses (e.g., sensitivity or subgroup analyses, meta-regression), if done, indicating which were pre-specified. | 7 |
| **RESULTS** | | |  |
| Study selection | 17 | Give numbers of studies screened, assessed for eligibility, and included in the review, with reasons for exclusions at each stage, ideally with a flow diagram. | 8 |
| Study characteristics | 18 | For each study, present characteristics for which data were extracted (e.g., study size, PICOS, follow-up period) and provide the citations. | Table 1 |
| Risk of bias within studies | 19 | Present data on risk of bias of each study and, if available, any outcome level assessment (see item 12). | 8 |
| Results of individual studies | 20 | For all outcomes considered (benefits or harms), present, for each study: (a) simple summary data for each intervention group (b) effect estimates and confidence intervals, ideally with a forest plot. | 8-14 |
| Synthesis of results | 21 | Present results of each meta-analysis done, including confidence intervals and measures of consistency. | 8-9 |
| Risk of bias across studies | 22 | Present results of any assessment of risk of bias across studies (see Item 15). | Sup. mat |
| Additional analysis | 23 | Give results of additional analyses, if done (e.g., sensitivity or subgroup analyses, meta-regression [see Item 16]). | N/A |
| **DISCUSSION** | | |  |
| Summary of evidence | 24 | Summarize the main findings including the strength of evidence for each main outcome; consider their relevance to key groups (e.g., healthcare providers, users, and policy makers). | 15-17 |
| Limitations | 25 | Discuss limitations at study and outcome level (e.g., risk of bias), and at review-level (e.g., incomplete retrieval of identified research, reporting bias). | 16-17 |
| Conclusions | 26 | Provide a general interpretation of the results in the context of other evidence, and implications for future research. | 17 |
| **FUNDING** | | |  |
| Funding | 27 | Describe sources of funding for the systematic review and other support (e.g., supply of data); role of funders for the systematic review. | 18 |

**Supplementary Item 2: Search Terms**

**MEDLINE**

(Nervous System Diseases [Mesh] or "nervous system" OR "stroke" OR "brain" OR "neuromuscular" OR "guillain-barre" OR "seizure" OR cerebrovasc* OR encephal* OR mening* OR demyelin* OR neurop* OR neurotrop* OR neuron*) AND ("covid-19" OR "sars-cov-2" OR "ncov-19" OR "ncov-2019" OR "covid 19" OR "sarscov2" OR "coronavirus") AND (presentati* OR manifest* OR complicat* OR symptom* OR "syndrome" OR "injury")

**Embase**

("nervous system" OR "stroke" OR "brain" OR "neuromuscular" OR "guillain-barre" OR "seizure" OR cerebrovasc* OR encephal* OR mening* OR demyelin* OR neurop* OR neurotrop* OR neuron*) AND ("covid-19" OR "sars-cov-2" OR "ncov-19" OR "ncov-2019" OR "covid 19" OR "sarscov2" OR "coronavirus") AND (presentati* OR manifest* OR complicat* OR symptom* OR "syndrome" OR "injury")

**Scopus**

("nervous system" OR "stroke" OR "brain" OR "neuromuscular" OR "guillain-barre" OR "seizure" OR cerebrovasc* OR encephal* OR mening* OR demyelin* OR neurop* OR neurotrop* OR neuron*) AND ("covid-19" OR "sars-cov-2" OR "ncov-19" OR "ncov-2019" OR "covid 19" OR "sarscov2" OR "coronavirus") AND (presentati* OR manifest* OR complicat* OR symptom* OR "syndrome" OR "injury")

**Supplementary Item 3: Case Reports**

| Study details | | Patient Demography | | | | Description of manifestation | | | | |
| --- | --- | --- | --- | --- | --- | --- | --- | --- | --- | --- |
| Author | **Date** | **No. patients in case Report** | **Patient Age** | **Sex** | **History/comorbidity** | **Presentation** | **Clinical features** | **Investigations** | **Management** | **Outcome** |
| INTRACRANIAL HAEMORRHAGE | | | | | | | | | | |
| Morassi[63] | 20/05/2020 | 2 | 57 | M | Arterial hypertension, thrombocytosis | Presented with 3 day history of fever, cough, and SOB. Confirmed SARS-COV-2 by nasopharyngeal swab. | Respiratory function deteriorated over 3 days post-admission warranting ICU admission and invasive ventilation. 11 days post-admission patient was found with a GCS of 3 and bilaterally fixed and dilated pupils. | Head CT identified large-diameter haemorrhages in both cerebral hemispheres. Supratentorial hydrocephalus and sulci obliteration were noted. | No management of neurologic injury. | Patient died 1-hour following the head CT. |
|  |  |  | 57 | M | No significant history | 10-day history of cough, fever and SOB confirmed to be SARS-COV-2. On Day 5 post-admission respiratory function worsened necessitating ventilation. | 7 days post-admission patient was found with GCS 3 with bilaterally fixed and dilated pupils. | Brain CT identified diffuse cerebral oedema with a large-volume haemorrhage in the right frontal lobe (6x5cm) extending to the ventricles. Multiple small haemorrhages were identified in both hemispheres. | No management of neurologic injury. | Patient died shortly after head CT. |
| Zahid[34] | 31/05/2020 | 1 | 38 | M | N.R. | Presented to ED with fever, cough and SOB. Respiratory function worsened leading to invasive ventilation & ICU admission. ECMO was commenced with continuous heparin infusion. | Neurologic examination during ICU stay identified right facial weakness and dysarthria. | Head CT showed a left-sided sub-insular parenchymal haemorrhage (2.6cm diameter). Continuous EEG monitoring showed generalised slowing with nil seizures. | N.R. | Resolution of bleed on follow-up CT with simultaneous improvement of symptoms. |
| Dixon[30] | 28/05/2020 | 1 | 59 | F | Aplastic anaemia managed with transfusions | Presented to emergency department with fleeting episodes of vacant staring, globalised tonic-clonic seizures, and vomiting. Had a 10-day history of persistent cough, sore throat, SOB, and headache. | Admission GCS was 11, vital signs normal. Neurologic examination initially identified nil focal deficits. Within 12 hours of admission GCS reduced to 5, positive Babinski sign, unreactive left pupil. | Nasopharyngeal swab for SARS-COV-2 was positive. CT identified brainstem swelling with intrinsic pontine haemorrhage and left posterior circulation occlusion. MRI at 6 days identified diffuse swelling and haemorrhage in the brain stem. | Patient was intubated and transferred to ICU. Imaging and neurologic progression led to diagnosis of haemorrhagic acute necrotizing encephalopathy. Withdrawal of ventilation at 10 days post ICU admission. | Patient died 10 days post admission. |
| Poyiadji[31] | 27/03/2020 | 1 | 58 | F | N.R. | 3-day history of cough, fever, and altered mental status. SARS-COV-2 nasopharyngeal RT-PCR was positive. | N.R. | Unenhanced head CT showed symmetric hypodensity of the thalami. MRI identified haemorrhagic rim-enhancing lesions within the thalami bilaterally, as well as lesions within the temporal lobes and subinsular regions. | IVIg was commenced. High-dose steroids contraindicated by active pneumonia. | N.R. |
| Kaya[33] | 2020 | 1 | 38 | M | N.R. | Patient presented to ED with a 5-day history of fever with nil other symptoms. | Bilateral adventitia noted on chest auscultation. Vital signs normal except fever. O2 saturation decreased to 88% on day-2 warranting intubation and ICU transfer. On day-5 of ICU stay, patient became acutely confused and agitated. Neurologic examination identified severe impairment of visual acuity with nil other deficits. | Brain MRI showed hyperintensities bilaterally, namely in the left occipital lobe and frontal cortical white matter tracts in the Corpus Callosum. DWI identified vasogenic oedema associated with posterior circulation. | Anti-viral therapy was ceased upon neurologic diagnosis with dexamethasone therapy commenced. | Patient neurologic examination improved after second dose of corticosteroids. Full recovery by the 10th day of hospital admission. |
| Franceschi[32] | 1/07/2020 | 2 | 48 | M | Obesity | Patient presented to ED with fever and cough with laboratory confirmed SARS-COV-2 infection. Patient transferred to ICU due to worsening pneumonia. | 2 weeks post-admission patient developed shock with large variability in blood pressure. Patient was diagnosed with cytokine release syndrome and developed an altered mental status. | Head CT identified focal vasogenic oedema in the posterior parieto-occipital regions of the brain bilaterally with small right-sided haemorrhage. Follow-up MRI at 1-month post-admission confirmed PRES and microhemorrhages on SWI. | Managed with antiviral therapy. Nil report of specific neurologic management. | Partial improvement in mental status at discharge (1 month). |
|  |  |  | 67 | F | Asthma, CAD, hypertension, T2DM and gout. | Patient presented to ED with lethargy and confusion. No respiratory symptoms noted. | Vital signs normal excepting labile blood pressure ranging from 120-170 systolic. | Blood analysis identified hyponatraemia and altered blood urea and creatinine. Nasopharyngeal SARS-COV-2 RT-PCR was positive. Head CT demonstrated bilateral oedema in the parieto-occipital regions with mass-effect and cortical sulcal effacement. MRI showed multiple oedematous regions and microhemorrhage in the posterior circulation. | Managed with antiviral therapy. Nil report of specific neurologic management. | Partial improvement in mental status at discharge. |
| ENCEPHALITIS | | | | | | | | | | |
| Ye[37] | 10/04/2020 | 1 | N.R. | M | N.R. | Patient presented to ED with a two week history of myalgia, SOB and fever. SARS-COV-2 was confirmed by nasopharyngeal swab. Patient developed impaired consciousness prompting neurologic admission. | Vital signs were stable. Nuchal rigidity, Kernig sign, and Brudzinski signs were positive. Extensor plantar reflex present. | CSF analysis was normal with negative RT-PCR for SARS-COV-2. | Based on clinical syndrome and concurrent pneumonia, neurologic team diagnosed with viral encephalitis. Mannitol infusion was commenced. | Consciousness completely improved within two weeks of treatment. |
| Moriguchi[36] | 25/03/2020 | 1 | 28 | M | N.R. | Presented to ED after being found unconscious on ground following a 9-day history of headache, fatigue, and coryzal symptoms. | GCS on admission was 6, with normal vital signs. Nuchal rigidity noted. | Blood tests identified elevated white cell count. CSF analysis was positive for SARS-COV-2 viral RNA using RT-PCR. Nasopharyngeal swab was negative. Brain DWI identified hyperintensity along the right lateral ventricle wall. FLAIR identified focal hyperintense signal changes in the right temporal lobe. | Patient was transferred to ICU for intubation and management of COVID-19 pneumonia. Diagnosis based on MRI was ventriculitis/encephalitis with a differential diagnosis of hippocampal sclerosis accompanying post-convulsive encephalopathy. | Still in intensive care unit at time of publication (day 15 post admission) |
| Wong[38] | 2020 | 1 | 40 | M | Hypertension and glaucoma | Presented to ED with 10-day history of persistent fever, worsening SOBOE, diarrhoea, and productive cough. | Patient was febrile with 93% O2 saturation on admission. No focal neurologic deficits. On day 3, patient developed unsteady gait followed by diplopia, oscillopsia, ataxia, altered sensation, and dysphagia. | Patient tested positive for SARS-COV-2 on RT-PCR. MRI revealed hyperintense lesions on T2 in the cervical spine and brainstem. Swelling and microhemorrhage were noted. CSF analysis yielded no findings. | Patient managed with anti-viral therapy for treatment of pneumonia. Patient commenced on gabapentin for management of ataxia and oscillopsia. | Patient's neurologic symptoms improved with discharge after 11 days. Gabapentin therapy continuing. |
| Al-olama[35] | 20/05/2020 | 1 | 36 | M | N.R. | Presented to ED with 6-day history of fever, headache, myalgia, cough, diarrhea, and vomiting. Previous COVID-19 test was negative. Patient had newly developed drowsiness and confusion. | GCS was 13 on admission with normal vitals. Patient was drowsy but rousable and oriented. Nil signs of meningitis. | Repeat SARS-COV-2 nasopharyngeal swab was positive. Head CT identified right-frontal intracerebral haematoma with associated subarachnoid haemorrhage. CT angiography ruled-out vascular aetiology, confirming viral encephalitis with haematoma formation. | Haematoma was surgically drained with fluid testing positive for SARS-COV-2 RNA on RT-PCR. COVID-19 managed with antiviral therapy. | N.R. |

| DELIRIUM | | | | | | | | | | | | | | | | | | | | | | | | | |
| --- | --- | --- | --- | --- | --- | --- | --- | --- | --- | --- | --- | --- | --- | --- | --- | --- | --- | --- | --- | --- | --- | --- | --- | --- | --- |
| Alkeridy[39] | | 10/05/2020 | | 1 | | 73 | | | M | T2DM, hypertension, IHD | | Patient presented to hospital by ambulance following fall at home. Presented with elevated BP (170/60mmHg), but normal HR, temperature and O2 saturation. Nil respiratory symptoms or fever noted | | Initially patient solely presented with confusion, inability to orient to person, and bilateral lower limb weakness. Patient developed fever 24 hours following admission, and cough 48 hours following admission. | | | Plain head-CT revealed no findings. Cerebral angiogram revealed no vascular disease. Chest CT at 48 hours consistent with COVID-19. | Patient administered a 10-day course of hydroxychloroquine and a 6-day course of piperacillin/tazobactam. | | | | | Patient responded to therapy with symptoms resolved by 8 days post-admission. Discharged after 13 days. | | |
| Butt[40] | | 2020 | | 1 | | 77 | | | M | Hypertension | | Patient presented to hospital following period of unusual behaviour and confusion. Nil respiratory symptoms were noted. | | Initial examination revealed inattention, disorientation, GCS 14, and normal O2 saturation. Neurologic examination unremarkable. Chest exam revealed bilateral crepitations in mid and basal areas. | | | Head CT revealed no findings. X-ray identified bilateral chest opacities consistent with pneumonia. Biochemistry identified elevated CRP and hyponatraemia. Oropharyngeal swab confirmed SARS-COV-2 infection. | Patient treated with antibiotics for 5 days post-admission and IV saline administered. | | | | | Patient's confusion remained for 4 weeks, during which a hospital-acquired infectiondeveloped. Patient died. | | |
| Soysal[41] | | 16/07/2020 | | 1 | | 70 | | | M | N.R. | | Patient presented with inattention, confusion, and behavioural abnormalities. No respiratory symptoms reported. | | Patient had no respiratory symptoms for duration of hospital stay, but manifested with fever shortly after admission to hospital. | | | Nasopharyngeal swab confirmed SARS-COV-2 infection with subsequent chest CT was consistent with COVID-19 pneumonia. | Treated with hydroxychloroquine, azithromycin, and Enfluvir. At 12 days, Tocilizumab therapy was commenced. Patient recovered from pneumonia with concurrent treatment at delirium clinic. | | | | | Discharged at 14 days post-admission. | | |
| \| SEIZURE \| \| --- \| | | | | | | | | | | | | | | | | | | | | | | | | | |
| Vollono[44] | | 2020 | | 1 | | 78 | | | F | Hypertension, postencephalitic epilepsy (2 years prior) | | Patient presented to emergency department with ongoing myoclonic jerks of the right face and limbs. 12 hours post-admission patient developed fever. | | Patient vital signs were normal. Neurologic examination identified fluent aphasia, right central facial nerve palsy, pronation of the right arm, and right leg drift. The patient had ongoing myoclonic jerks of the right eyelid and lip. Symptoms persisted for >2.5 hours leading to status epilepticus diagnosis. | | | EEG revealed semi-rhythmic, irregular, high-amplitude delta activity lateralised to the left motor regions. Head CT showed nil findings. Brain MRI confirmed gliosis and atrophy of the left temporo-parietal lobe. Nasopharyngeal swab positive for SARS-COV-2 viral RNA. | Treated with antiepileptics and antiviral therapy. | | | | | SE responded to antiepileptics and illness resolved. Discharged 2 weeks post-admission. | | |
| Fasano[43] | | 15/4/2020 | | 1 | | 54 | | | M | No significant medical history. | | Patient presented to ED following single clonic seizure affecting the right arm. The patient lost consciousness. The seizure stopped after 2 minutes followed by a 30-minute period of confusion. Patient reported a 10-day history of fever and conjunctivitis. | | Vital signs normal on admission. Neurological examination revealed nil findings. | | | Head CT, serum analysis, and urine analysis were normal. | Patient initially discharged but re-presented 4 days later with worsening of respiratory condition which was diagnosed as SARS-COV-2. Admitted to ICU and treated with antiviral therapy | | | | | Discharged after two weeks of therapy. | | |
| ACUTE DISSEMINATED ENCEPHALOMYELITIS | | | | | | | | | | | | | | | | | | | | | | | | | |
| Reichard[46] | | 7/07/2020 | | 1 | | 71 | | M | Hx of IHD | | | Patient underwent CABG due to worsening of coronary artery disease. Tested positive for SARS-COV-2 6-days post-op. | | Patient required intubation/sedation with worsening respiratory and kidney failure. Died 11-days post-op. | | | Post-mortem examination revealed multiple intracerebral haemorrhagic lesions ranging from 1mm to 1cm, as well as multiple inflammatory demyelinating lesions in the sub-cortical white matter. | | | | No management of neurologic symptoms (diagnosed postmortem). | | Died in ICU | | |
| Novi[45] | | 3/06/2020 | | 1 | | 64 | | F | Hypertension, monoclonal gammopathy of undetermined significance | | | Patient experienced a two-week flu-like illness which was confirmed with serum IgG to be COVID-19. Approximately 3-4 weeks later she presented to hospital with bilateral visual impairment. | | Neurological exam revealed mild behavioural abnormalities, RAPD, headache, anosmia, poor visual acuity (hand motion), lower limb hyperreflexia and Babinski sign. | | MRI revealed multiple T1 post-Gadolinium enhancing lesions in the spinal cord and optic nerves. Lumbar puncture showed lymphocytic pleocytosis. rtPCR was positive for SARS-COV-2 in the CSF. | | | | ADEM disease was suspected, commenced on high-dose IV methylprednisolone and IV immunoglobulins. | | Patient recovered visual acuity after two weeks of treatment and subsequent MRI identified reduced number of lesions. Discharged from hospital. | | |  |
| GUILLAIN-BARRE SYNDROME | | | | | | | | | | | | | | | | | | | | | | | | |  |
| Sedaghat[64] | | 11/04/2020 | | 1 | | 65 | | M | T2DM | | | Patient had a 5-day history of acute progressive weakness of the lower extremities progressing into proximal weakness and quadriplegia at the time of presentation. Two weeks prior to admission patient was diagnosed with COVID-19 by nasopharyngeal swab + CT | | Patient presented with quadriplegia, absent reflexes and bilateral facial paresis. Afebrile, with normal BP, HR, and O2 saturation. Patient was conscious and had no dyspnoea or respiratory distress. | | Biochemistry yielded nil significant findings, nor did brain or spine MRI. Lung CT showed diffuse ground glass opacities and bilateral pleural effusion. Neurophysiology studies consistent with GBS; decreased recruitment on electromyography, decreased amplitude at compound muscle action and nil sensory response. | | | | Managed with 5-day course of intravenous immunoglobulin. | | Not reported. | | |  |
| Assini[65] | | 28/05/2020 | | 2 | | 55 | | M | N.R. | | | Hospitalised for severe respiratory syndrome on a background of anosmia, ageusia, and cough. After 3 days the patient was intubated for mechanical ventilation. 20 days post-admission patient developed bilateral eyelid ptosis, dysphagia, and dysphonia. | | Neurologic exam identified bilateral facial paresis, hypoglossal nerve dysfunction, 10th CN motor dysfunction, and hyporeflexia of the upper limbs. No muscle weakness noted. | | Nil findings on brain MRI. CSF and serum examination identified oligoclonal bands and increased IgG/albumin ratio. CSF RT-PCR was negative. Electroneurography identified bilateral demyelination with sural sparing. | | | | Patient treated with IV IG (0.4g/kg/day) with improvement after 5 days. | | Patient fully recovered, nil timeframe reported. | | |  |
|  | |  | |  | | 60 | | M | N.R. | | | Patient presented to hospital with fever and cough and was diagnosed with COVID-19. Three days later, respiratory function worsened necessitating tracheostomy and ventilation. 20 days later patient developed distal weakness in lower limbs. | | Neurological examination identified decreased power in both lower limbs with arreflexia and unilateral footdrop on right side. Gastroplegia, paralytic ileus, and autonomic dysregulation were also noted. | | Electroneurography identified sensory-motor axonal polyneuropathy. EMG showed neurogenic changes in all muscles. Oligoclonal bands identified in CSF and serum, with elevated IgG/albumin ratio in CSF. CSF RT-PCR negative. | | | | Patient treated with IV IG (0.4g/kg/day) with improvement after 5 days. | | Partial recovery with persistence of hyporeflexia and right foot drop. | | |  |
| Tiet[66] | | 1/07/2020 | | 1 | | 49 | | M | No significant history. | | | Patient presented with 3 weeks SOB. Afebrile with worsening cough and distal lower limb paraesthesia affecting mobilisation. Oropharyngeal swab confirmed SARS-COV-2. Patient initially discharged with diagnosis of viral myositis. Re-presented 3 days later with worsening lower limb paraesthesia and ascending lower limb weakness. Four days later developed facial diplegia, weakness, and muscle weakness in all limbs. | | Initial examination showed mild tachycardia (109), normal blood pressure, and normal O2 saturation. Neurologic examination revealed reduced sensation and areflexia in distal limbs. Developed dysesthesia in lower limbs. | | CSF showed cytoalbuminologic dissociation. CSV RT-PCR negative. Neurophysiology revealed absent sensory response of median nerve and severe slowing of motor responses in median & ulnar nerve. Demyelinating polyneuropathy identified meeting Brighton criteria for diagnosis of GBS. | | | | Patient treated with IV IG (0.4g/kg/day) with improvement within first week. | | Gross neurologic deficits recovered after four weeks, with unassisted mobilisation possible after 11 weeks of neurorehabilitation. | | |  |
| Scheidl[67] | | 1/05/2020 | | 1 | | 54 | | F | No significant history. | | | Patient was admitted to neurology department with a 10-day history of worsening symmetric lower limb weakness. She tested positive for SARS-COV-2 on nasopharyngeal swab 3 weeks earlier after a close contact with an infected person, but did not have any coryzal symptoms except loss of smell. | | Proximally pronounced, moderate, symmetric parapaeresis was reported with numbness and tingling of the extremities. Neurologic examination identified areflexia, normal gait, and decreased proximal and distal muscle power. Two-days post admission patient reported dysphagia. | | Follow-up nasopharyngeal RT-PCR was negative for SARS-COV-2. CSF studies identified albuminocytogenic dissociation with increased protein level. Serum testing and MRI of the cervical spine yielded no findings. Electrophysiology studies identified prolonged distal muscle latencies. Denervation wasn't identified on EMG. | | | | Patient diagnosed with AIDP type GBS, treated with IV IG (0.4g/kg/day) with improvement in first 5 days. | | Patient made full recovery. | | |  |
| Caamano[68] | | 4/05/2020 | | 1 | | 61 | | M | N.R. | | | Patient was diagnosed with SARS-COV-2 pneumonia after presenting with fever and cough. Pneumonia mostly resolved after 1 week. On day 10 of admission, patient developed neurologic symptoms. | | Patient noted liquid dripping from right side of mouth. Right facial nerve palsy developed, followed by bilateral progression. Neurologic exam revealed nil findings excepting bilateral facial nerve palsy as indicated by unresponsive blink reflexes and muscle paresis. | | Lumbar puncture revealed elevated protein, absent leukocytes, and negative RT-PCR. Brain CT and MRI revealed nil pathologic findings. | | | | Low-dose oral prednisolone administered. | | Slight improvement after two weeks of therapy. Partial recovery. | | |  |
| Coen[69] | | 28/04/2020 | | 1 | | 70s* | | M | No significant history. | | | Patient admitted to hospital with a 10-day history of myalgia, fatigue, and dry cough. RT-PCR confirmed SARS-COV-2 infection. | | Examination on admission revealed bilateral lower limb flaccid paresis, absent tendon reflexes, and indifferent plantar reflexes. Nil sensory deficit identified. | | CSF showed cytoalbuminologic dissociation. CSV RT-PCR negative. Neurophysiology revealed sensorimotor demyelinating polyneuropathy with sural sparing. | | | | Patient commenced on IV IG (0.4g/kg/day) 1 day following admission. Patient responded to treatment within days. | | Patient transferred to neurorehabilitation. Follow-up serology confirmed SARS-COV-2 infection. | | |  |
| Farzi[70] | | 16/06/2020 | | 1 | | 41 | | M | T2DM | | | Patient diagnosed with COVID-19 pneumonia on admission. 10 days post-admission and treatment with antiviral medication, patient developed neurologic symptoms. | | Patient experienced paraesthesia in the distal lower limbs which progressed to proximal muscle weakness. Seven days following onset of neurologic symptoms patient was unable to mobilise. Neurologic examination revealed absent tendon reflexes, symmetric weakness in upper and lower limbs, and glove-and-stocking hypesthesia. | | EMG revealed absent right tibial and bilateral peroneal nerve CMAPs. All SNAP latencies were prolonged. | | | | Patient commenced on IV IG (0.4g/kg/day). Patient started responding to treatment within 3 days. | | Partial recovery with residual weakness in lower extremities. | | |  |
| Orguz-Akarsu[49] | | 2020 | | 1 | | 53 | | F | No significant history. | | | Patient presented with a 3-day history of dysarthria with progressive weakness and numbness of the lower extremities. | | Neurologic examination revealed mild dysarthria due to jaw weakness and symmetrical bilateral lower limb weakness. Patient could only mobilise with assistance. Tendon reflexes absent in lower extremities | | Biochemical screening and CSF analysis revealed nil findings. Nerve conduction studies confirmed a demyelinating syndrome. MRI identified asymmetrical thickening and hyperintensity of post-ganglionic roots using short-tau inversion recovery (STIR). Nasopharyngeal swab positive for SARS-COV-2 | | | | Patient treated with plasma exchange after 5 days of onset of symptoms. | | Patient markedly improved after 2 weeks of treatment. | | |  |
| Rana/Virani[71,72] | | 2020 | | 1 | | 54 | | M | HTN, hyperlipidaemia, chronic LBP, restless leg syndrome | | | Patient presented with progressive shortness of breath following a 2-week period of coryzal symptoms which had resolved. Patient required intubation and ventilation soon after admission. His wife had tested positive for COVID-19 and his symptoms started soon after. | | Neurological examination on admission revealed quadriparesis and areflexia with mute plantar responses. | | COVID-19 nasopharyngeal swab was positive. MRI revealed nil brain or spine findings. Lumbar puncture was deferred. EMG confirmed demyelinating syndrome consistent with Miller-Fisher type GBS. | | | | Patient commenced on IV IG (0.4g/kg/day). Patient started responding to treatment within 3 days. Respiratory function improved following antiviral therapy for COVID-19. | | Partial recovery necessitating ongoing neurorehabilitation. | | |  |
| Ottaviani[73] | | 12/05/2020 | | 1 | | 66 | | F | HTN | | | Patient presented with 72-hour history of increasing difficulty walking on a 10-day background of ever and cough. | | Transient pruritic dorsal rash on admission. Examination identified paraparesis with rapidly progressive symmetric weakness in lower limbs and absent tendon reflexes. Nil sensory deficits noted. | | Lumbar puncture demonstrated albumin-cytological dissociation. EMG post-IV-IG therapy was consistent with mixed demyelination and axonal damage. Concurrently, patient tested positive for SARS-COV-2 on nasopharyngeal RT-PCR. | | | | IV-IG commenced (0.4g/kg/day) for 5 days. | | Patient required ICU admission for multi-organ failure due to respiratory syndrome. Neurologic symptoms worsened and did not respond to therapy. | | |  |
| Padroni[74] | | 17/04/2020 | | 1 | | 70 | | F | N.R. | | | Patient presented to ED complaining of asthenia, glove-and-stocking paraesthesia and difficulty mobilising. 3 weeks earlier patient experienced fever and dry cough which resoled in a few days, and tested positive for SARS-COV-2. | | Neurologic examination identified moderate symmetrical distal upper and lower limb weakness, areflexia, and preserved sensation. | | Serum biochemistry yielded nil findings. CSF analysis identified albumino-cytological dissociation. | | | | IV-IG commenced (0.4g/kg/day) for 5 days. After 3 days, patient required intubation and mechanical ventilation due to worsening muscle weakness. | | Not reported. | | |  |
| Toscano[47] | | 25/06/2020 | | 5 | | 77 | | F | N.R. | | | Patient presented to ED with rapidly progressing paraesthesia in the lower limbs and hands. This occurred 7 days after development of cough and fever. Nasopharyngeal swab confirmed SARS-COV-2 infection | | Neurologic examination identified flaccid areflexic tetraplegia. | | Chest CT identified interstitial pneumonia consistent with COVID-19. EMG identified an axonal variant of GBS with sural sparing. Bulbar symptoms developed during IVIG treatment necessitating non-invasive ventilation. | | | | Patient treated with IVIG. Plasmapheresis contraindicated by active infection. | | Patient showed minimal improvement to first course of IVIG and was unresponsive to second course. | | |  |
|  | |  | |  | | 23 | | M | N.R. | | | Patient presented to ED complaining of upper and lower facial weakness which progressed over two days. Patient also noted mastoid pain, loss of taste, and lower limb paraesthesia. This occurred 10 days after fever and sore throat which was managed with AB. | | Neurological examination revealed complete facial palsy, generalised areflexia, and sensory ataxia. | | Brain MRI revealed focal contrast enhancement of internal acoustic meatus. EMG confirmed axonal damage with sural sparing consistent with GBS. RT-PCR of nasopharyngeal swab was positive for SARS-COV-2. | | | | Patient received course of IVIg. | | Patient's limb paresthesia completely resolved, with incomplete resolution of facial weakness. | | |  |
|  | |  | |  | | 55 | | M | N.R. | | | Patient presented to ED with fever and cough. COVID-19 was confirmed. 10 days later, patient developed neurologic symptoms. | | Patient reported neck pain, tingling in all limbs, and lower limb weakness. Patient developed facial diplegia and respiratory failure during IVIg treatment. | | EMG identified severe axonal neuropathy. | | | | Patient received two courses of IVIg therapy and was admitted to ICU due to respiratory failure caused by neuromuscular dysfunction. | | Patient unresponsive to treatment. Remained in ICU at time of publishing (30-days post-admission). | | |  |
|  | |  | |  | | 76 | | M | N.R. | | | Patient presented with 5 day history of dry cough and loss of smell. Referred to neurosurgery due to lumbar pain and lower limb weakness. 4 days post admission patient developed worsening neurologic symptoms. | | Neurologic examination identified progression of muscle weakness to flaccid areflexic tetraparesis. Patient then developed fever following onset of neurologic symptoms, and tested positive to SARS-COV-2 on nasopharyngeal swab. | | Chest imaging identified no abnormalities. | | | | Patient received 5-day course of IVIG (0.4g/kg/day). | | Partial recovery of motor function in upper limbs. Still unable to stand upon referral to neurorehab. | | |  |
|  | |  | |  | | 61 | | M | N.R. | | | Patient presented to hospital with a 2- week history of asthenia, dry cough, anosmia, and no fever. Patient developed lower limb paraesthesia and difficulty climbing stairs in the week preceding admission. | | Neurological exam revealed generalised areflexia and paraparesis. | | Chest imaging identified interstitial pneumonia consistent with COVID-19. Nasopharyngeal swab was negative for SARS-COV-2. CSF analysis was normal. EMG at 4 days post-admission showed conduction block and demyelination consistent with GBS. Serum testing after the patient was discharged identified SARS-COV-2 IgG. | | | | IVIG was commenced. After two days, patient developed flaccid tetraplegia and dysphagia. The next day the patient was admitted to ICU and mechanical invasive ventilation was commenced due to neuromuscular failure. Patient developed Acinetobacter pneumonia during ventilation which was managed with AB. Plasmapheresis was commenced after resolution of pneumonia. | | Still in ICU receiving mechanical ventilation. | | |  |
| Zhao[75] | | 1/04/2020 | | 1 | | 61 | | F | N.R. | | | Presented with acute weakness in both legs and severe fatigue. No history of cough, fever, or diarrhea. | | Normal vital signs on admission. Lung auscultation showed no abnormalities. Neurologic examination identified symmetric bilateral lower limb weakness and areflexia. Over 3 days upper limb weakness also developed, as well as glove-and-stocking sensory deficits. | | Serum analysis identified lymphocytopenia and thrombocytopenia. CSF identified elevated protein and normal cell counts. Neurophysiology studies identified demyelinating neuropathy consistent with GBS. | | | | Patient was commenced on IVIG (0.4g/kg/day). 8 days post-admission patient developed cough and tested positive for SARS-COV-2 on RT-PCR of nasopharyngeal swab. | | Resolution of neurologic symptoms by discharge (day 30). | | |  |
| Gutierrez[76] | | 1/01/2020 | | 2 | | 50 | | M | Bronchial asthma | | | Patient presented to hospital with a 2-day history of vertical diplopia, perioral paraesthesia, and gait instability. Patient had coryzal illness 5 days earlier. | | Neurologic examination revealed nil perioral or peripheral muscle weakness or sensory deficits. A broad ataxic gait was identified but no dysmetria. Deep tendon reflexes were absent in the upper and lower limbs. Right hypertropia was noted in all fields of gaze, with severe limitation of adduction and downgaze in the right eye, and left nystagmus. | | Serum analysis revealed lymphopenia and elevated CRP. Positive anti-GQ1b IgG. Oro-pharyngeal RT-PCR confirmed SARS-COV-2 infection. CSF analysis was normal. Chest x-ray and head CT were normal. | | | | Right internuclear ophthalmo-paresis was diagnosed, along with right oculomotor palsy. Patient managed with IVIG for 5 days. | | Nearly full recovery after two weeks with slight residual anosmia and ageusia. | | |  |
|  | |  | |  | | 39 | | M | Nil significant history. | | | Patient initially presented with diarrhea and fever with nil respiratory symptoms. Three days later patient developed diplopia. | | Neurologic exam identified slightly reduced visual acuity, severe abduction deficits in both eyes, and fixation nystagmus. All deep tendon reflexes were absent. No muscle weakness, sensory deficit, ataxia or dysmetria were identified. | | Chest x-ray and head CT were normal. Serum analysis identified leukopenia. RT-PCR of nasopharyngeal swab for SARS-COV-2 was positive. | | | | Patient treated symptomaticaly with follow-up telehealth consultation. | | 2 weeks later the patient had fully recovered with no residual neurologic deficits. | | |  |
| Lantos[48] | | 1/07/2020 | | 1 | | 36 | | M | Childhood left eye strabismus. | | | Presented to ED with left eye drooping, blurry vision, reduced sensation and paraesthesia in both legs. Patient had a 4-day history of coryzal symptoms, coming from a COVID-19 endemic area. | | Examination identified partial left third nerve palsy and decreased sensation in the distal lower limbs (all modalities). | | MRI identified enlargement and gadolinium enhancement of the left CN3. No other findings. RT-PCR confirmed COVID-19. | | | | Patient was treated with IVIG and antiviral therapy. | | Patient discharged after 4 days with recovery of left eye function motor. | | |  |
| OTHER PERIPHERAL NEUROPATHY | | | | | | | | | | | | | | | | | | | | | | | | |  |
| Abdelnour[50] | | 1/04/2020 | | 1 | | 69 | | M | HTN, T2DM, mild COPD | | | Presented with 3-day history of bilateral lower limb weakness. Nil respiratory symptoms. Patient has background of chronic cough. | | Neurologic examination showed reduced power to 4/5 in knee extension with normal power of all other muscles. Deep tendon reflexes in the lower limbs were absent, and an ataxic gait was noted. On day 4 post-admission, patient developed fever and confusion. Nasopharyngeal SARS-COV-2 RT-PCR was positive | | MRI of head and whole spine revealed no evidence of inflammation or demyelination. Evidence of chronic infarcts throughout the left hemisphere. | | | | No specific management administered. No specific diagnosis made, solely a motor neuropathy affecting the lower limbs. | | Progressive spontaneous recovery with discharge at 18 days. | | |  |
| Dinkin[51] | | 4/08/2020 | | 2 | | 36 | | M | Infantile strabismus | | | Presented to ED with left ptosis, diplopia, bilateral leg paraesthesia. Fever, cough, and myalgia developed over 4 days earlier but had resolved. | | Neurologic examination revealed left mydriasis, ptosis, and impaired depression and adduction, indicating a left oculomotor nerve palsy. Bilateral abducens palsy also suspected. Lower limb hyporeflexia, hypesthesia, and gait ataxia were noted. | | Nasopharyngeal SARS-COV-2 RT-PCR was positive. MRI revealed T2 enhancement of the left oculomotor nerve. | | | | Patient treated with IVIg | | Symptoms partially resolved 3 days post admission. | | |  |
|  | |  | |  | | 71 | | F | HTN | | | Patient presented with 2 day history of painless diplopia and failure of right-sided eye abduction. Patient was febrile and SOB. | | Neurologic exam found normal visual acuity, pupillary reactivity, and fundoscopy. | | Patient tested positive for SARS-COV-2. | | | | No specific management of neurologic symptoms. | | Patient discharged with minimal recovery. Reported partial recovery via telephone follow-up 2 weeks later | | |  |
| Goh[52] | | 21/05/2020 | | 1 | | 27 | | M | None | | | Presented to ED with myalgia, cough, and fever over 4 days. Patient had a new left-sided headache. | | Mild bilateral conjunctival injection was noted. No abnormalities on respiratory or neurologic examination. 3 days post-admission, patient developed left-sided facial weakness preceded by retro-auricular pain and dysgeusia. Neurologic exam confirmed facial nerve palsy. | | Positive nasopharyngeal swab for SARS-COV-2. No abnormalities on CSF analysis. MRI showed enhancement of the left facial nerve. | | | | Provisional diagnosis of Bell's palsy treated with prednisone and valacyclovir. Concurrent antiviral therapy for COVID-19. | | Headache and retro-auricular pain resolved in one week with minimal improvement in facial nerve palsy. | | |  |

**Supplementary Item 4: Quality assessment**

Case reports (based on modified Newcastle-Ottawa Score by Murad and colleagues). Quality score: Low = 0-2, Medium = 3-5, High = 6-8

| Author | Date | Title | Quality |
| --- | --- | --- | --- |
| Abdelnour | 2020 | COVID 19 infection presenting as motor peripheral neuropathy | Medium |
| Alkeridy | 2020 | A Unique Presentation of Delirium in a Patient with Otherwise Asymptomatic COVID-19 | Low |
| Al-olama | 2020 | COVID-19-Associated meningoencephalitis complicated with intracranial haemorrhage | Low |
| Ashrafi | 2020 | COVID-19-related strokes in adults below 55 years of age: a case series | Low |
| Assini | 2020 | New clinical manifestation of COVID-19 related Guillain-Barrè syndrome highly responsive to intravenous immunoglobulins: two Italian cases | Low |
| Avula | 2020 | COVID-19 presenting as stroke | Low |
| Butt | 2020 | Prolonged Confusional state as first manifestation of COVID-19 | Medium |
| Cariddi | 2020 | Reversible Encephalopathy Syndrome (PRES) in a COVID-19 patient | Medium |
| Chen | 2020 | COVID-19 with acute cerebral infarction: one case report | Medium |
| Chibane | 2020 | Hyperacute multi‑organ thromboembolic storm in COVID‑19: a case report | Low |
| Co | 2020 | Intravenous Thrombolysis for Stroke in a COVID-19 Positive Filipino Patient, a Case Report | Low |
| Coen | 2020 | Guillain-barre syndrome as a complication of SARS-COV-2 infection | Medium |
| Dinkin | 2020 | COVID-19 presenting with ophthalmoparesis from cranial nerve palsy | Low |
| Dixon | 2020 | COVID-19-related acute necrotizing encephalopathy with brain stem involvement in a patient with aplastic anemia | Low |
| Farzi | 2020 | Guillain-barre syndrome in a patient infected with SARS-COV-2, a case report | Low |
| Fasano | 2020 | First motor seizure as presenting symptom of SARS-CoV-2 infection | Medium |
| Franceschi | 2020 | Hemorrhagic Posterior Reversible Encephalopathy Syndrome as a Manifestation of COVID-19 Infection | Medium |
| Goh | 2020 | Pearls & Oy-sters: Facial Nerve Palsy as a Neurological Manifestation of Covid-19 Infection | Low |
| Griffin | 2020 | Arterial thromboembolic complications in COVID-19 in low-risk patients despite prophylaxis | Medium |
| Guilan | 2020 | Unusual simultaneous cerebral infarcts in multiple arterial territories in a COVID-19 patient | Medium |
| Gutierrez | 2020 | Miller Fisher syndrome and polyneuritis carnialis in COVID-19 | Medium |
| He | 2020 | Diagnosis and treatment of an elderly patient with secondary cerebral infarction caused by COVID-19 | Low |
| Hughes | 2020 | Cerebral Venous Sinus Thrombosis as a Presentation of COVID-19 | Low |
| Kaya | 2020 | Transient cortical blindness in COVID-19 pneumonia; a PRES-like syndrome: Case report | Medium |
| Lantos | 2020 | COVID-19 associated Miller Fisher Syndrome: MRI Findings | Low |
| Malentacchi | 2020 | Concomitant brain arterial and venous thrombosis in a COVID-19 patient | Medium |
| Morassi | 2020 | Stroke in patients with SARS‑CoV‑2 infection: case series | Medium |
| Moriguchi | 2020 | A first case of meningitis/encephalitis associated with SARS-Coronavirus-2 | Low |
| Novi | 2020 | Acute disseminated encephalomyelitis after SARS-CoV-2 infection | Low |
| Orguz-Akarsu | 2020 | Guillain-barre syndrome in a patient with minimal symptoms of COVID-19 infection | Medium |
| Ottaviani | 2020 | Early Guillain-Barre Syndrome in coronavirus disease 2019 (covid-19): a case report from an italian COVID-hospital | Low |
| Oxley | 2020 | Large-Vessel Stroke as a Presenting Feature of Covid-19 in the Young | Medium |
| Padroni | 2020 | Guillain-barre syndrome following covid-19: new infection, old complication? | Low |
| Rana | 2020 | Novel Coronavirus (COVID-19)-associated Guillain-barre syndrome: case report | Medium |
| Reddy | 2020 | Cerebrovascular Disease in Patients with COVID-19: A Review of the Literature and Case Series | Low |
| Reichar | 2020 | Neuropathology of COVID-19: a spectrum of vascular and acute disseminated encephalomyelitis (ADEM)-like pathology | Medium |
| Salomon | 2020 | Facial diplegia, a possible atypical variant of Guillain-barre syndrome as a rare neurological complication of SARS-COV-2 | Medium |
| Scheidl | 2020 | Guillain-Barre Syndrome during SARS-COV-2 panedmic: A case report and review of recent literature. | Medium |
| Sedaghat | 2020 | Guillain Barre syndrome associated with COVID-19 infection: A case report | Low |
| Sharifi-Razavi | 2020 | Ischemic stroke associated with novel coronavirus 2019: a report of three cases | Low |
| Soysal | 2020 | Delirium as the first clinical presentation of the coronavirus disease 2019 in an older adult | Low |
| Tiet | 2020 | Guillain-Barre Syndrome associated with COVID-19 infection: a case from the UK | Low |
| Toscano | 2020 | Guillain-Barre Syndrome associated with SARS-COV-2 | Medium |
| Tunc | 2020 | Coexistence of COVID-19 and acute ischemic stroke report of four cases | Medium |
| Vollono | 2020 | Focal status epilepticus as unique clinical feature of COVID-19: A case report | Medium |
| Wang | 2020 | Stroke and mechanical thrombectomy in patients with COVID-19: technical observations and patient characteristics | Low |
| Wong | 2020 | Lessons of the month 1: A case of rhombencephalitis as a rare complication of acute COVID-19 infection | Medium |
| Ye | 2020 | Encephalitis as a clinical manifestation of COVID-19 | Medium |
| Zahid | 2020 | Hemorrhagic Stroke in Setting of Severe COVID-19 Infection Requiring Extracorporeal Membrane Oxygenation (ECMO) | Low |
| Zayet | 2020 | Acute Cerebral Stroke with Multiple Infarctions and COVID-19, France 2020 | Low |
| Zhai | 2020 | The impact of COVID-19 on ischemic stroke | Medium |
| Zhao | 2020 | Guillain-Barre syndrome associated with SARS-COV-2 infection: causality or coincidence | Medium |
| Zhou | 2020 | A Case of Coronavirus Disease 2019 With Concomitant Acute Cerebral Infarction and Deep Vein Thrombosis | Low |

Cohort Studies (based on modified Newcastle-Ottawa Score by Rogers and colleagues). Quality score: Low = 0-3, Medium = 4-6, High = 7-9

| Author | Date | Title | Quality |
| --- | --- | --- | --- |
| Abdel-mannan | 2020 | Neurologic and Radiographic Findings Associated With COVID-19 Infection in Children | Medium |
| Helm* | 2020 | High risk of thrombosis in patients with severe SARS‑CoV‑2 infection: a multicenter prospective cohort study | Medium |
| Helm* | 2020 | Neurologic Features in Severe SARS-CoV-2 Infection | Medium |
| Jain | 2020 | COVID-19 related neuroimaging findings: A signal of thromboembolic complications and a strong prognostic marker of poor patient outcome | Medium |
| Klok | 2020 | Confirmation of the high cumulative incidence of thrombotic complications in critically ill ICU patients with COVID-19: An updated analysis | Medium |
| Kremer | 2020 | Brain MRI Findings in Severe COVID-19: A Retrospective Observational Study | Low |
| Li/Mao | 2020 | Acute cerebrovascular disease following COVID-19: a single center, retrospective, observational study | Low |
| Lodigiani | 2020 | Venous and arterial thromboembolic complications in COVID-19 patients admitted to an academic hospital in Milan, Italy | Medium |
| Lu** | 2020 | New onset acute symptomatic seizure and risk factors in coronavirus disease 2019: A retrospective multicenter study | Low |
| Merkler | 2020 | Risk of Ischemic Stroke in Patients with Covid-19 versus Patients with Influenza | Medium |
| Romero-Sanchez | 2020 | Neurologic manifestations in hospitalized patients with COVID-19: The ALBACOVID registry | Medium |
| Scullen | 2020 | Coronavirus 2019 (COVID-19) - Associated Encephalopathies and Cerebrovascular Disease: The New Orleans Experience | Medium |
| Vartharaj | 2020 | Neurological and neuropsychiatric complications of COVID-19 in 153 patients: a UK-wide surveillance study | High |
| Xiong** | 2020 | New onset neurologic events in people with COVID-19 infection in three regions in China | Low |
